# Multifunctional hydrogel sensors with dynamic covalent networks for machine learning-assisted Parkinson's disease diagnosis and encrypted human-computer interaction

**DOI:** 10.1016/j.mtbio.2025.102524

**Published:** 2025-11-04

**Authors:** Siqi Ding, Xiao Yu, Qi Wang, Peng Luo, Hua Li, Zhengrui Li, Ruhan Wang, Hengrui Liu, Yucang He, Jinyao Nong, Chao Zhang

**Affiliations:** a Department of Neurology, The Affiliated Yiwu Hospital of Wenzhou Medical University, 699 Jiangdong Road, Yiwu City, Zhejiang Province, 322000, China; b Department of Otolaryngology-Head and Neck Surgery, Shanghai Sixth People's Hospital, Affiliated to Shanghai Jiao Tong University School of Medicine, Shanghai, 200030, China; c Department of Oncology, Ruijin Hospital, Shanghai Jiao Tong University School of Medicine, Shanghai, 200025, China; d Southern Medical University, Guangzhou, Guangdong, China; e Life Science and Clinical Medicine Research Center, Affiliated Hospital of Youjiang Medical University for Nationalities, Baise, 533000, Guangxi, China; f Department of Neurosurgery, Qilu Hospital of Shandong University, China; g Department of Biochemistry, University of Cambridge, Cambridge, UK; h Department of Plastic Surgery, First Affiliated Hospital of Wenzhou Medical University, Nanbaixiang, Ouhai Direct, Wenzhou City, Zhejiang Province, 325000, China; i Department of Rehabilitation Medicine, Affiliated Hospital of Youjiang Medical University for Nationalities, Baise, 533000, Guangxi, China

**Keywords:** Hydrogel, Strain sensor, Human-computer interaction, Parkinson's, Smart healthcare

## Abstract

Flexible sensors hold significant application value in the fields of human-computer interaction and medical monitoring. In this study, we developed a smart hydrogel strain sensor based on dynamic covalent cross-linking. A multifunctional hydrogel was fabricated by constructing a multicomponent synergistic network composed of poly (acrylic acid) (PAA), dialdehyde carboxymethyl cellulose (OCMC), gelatin methacryloyl (GelMA), and lignosulfonate methacrylate (MLS). This hydrogel (AGOM) exhibited synergistically enhanced interfacial adhesion strength (>77.8 kPa) and mechanical properties, with an elongation at break exceeding 1820 %. The unique gradient network structure not only provides the material with excellent electrical conductivity (conductivity >0.55 S/m). A message transmission system has been developed based on the principles of Morse code, allowing for the coding and decoding of messages by recognizing different amplitudes of finger bending. Electrodes constructed from this hydrogel reliably record human electromyography (EMG) and electrocardiography (ECG) signals. The conductive network, combined with its wide-range and high-sensitivity characteristics (GF = 5.31), enables the sensor to accurately recognize clinical symptoms. This includes the characteristic tremor signals associated with Parkinson's disease. By leveraging machine learning, the sensor achieves a high recognition rate. This technology offers an innovative solution for monitoring and assisted treatment of Parkinson's disease, presenting significant application prospects in the field of intelligent medicine.

## Highlights

• A smart hydrogel strain sensor based on dynamic covalent cross-linking developed by component synergy network.
• Hydrogels have excellent bacteriostatic and biological properties and can be applied to physiological signal monitoring.
• This sensor recognizes the symptoms of Parkinson's disease and achieves high recognition rates through machine learning.

## Graphical abstract

**Figure:**
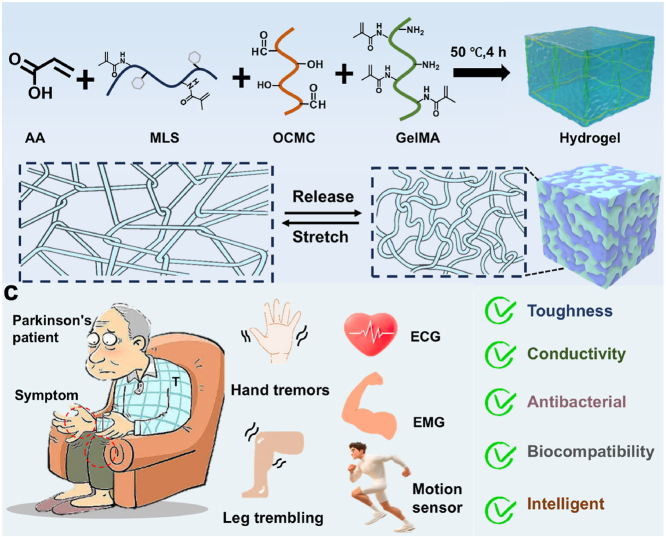

## 1 Introduction

In recent years, the rapid development of flexible electronics technology has led to revolutionary breakthroughs in physiological signal monitoring [[1], [2], [3]]. Notably, the innovative design of bionic-type flexible sensing systems offers a novel solution for real-time and accurate monitoring of human physiological states [[4], [5], [6], [7]]. Flexible electronics have emerged as transformative tools in medical monitoring and human-machine interfaces, providing unprecedented capabilities for real-time physiological signal acquisition and analysis [8,9]. One of the most promising applications lies in the management of neurodegenerative diseases, particularly Parkinson's disease (PD), where continuous monitoring of motor symptoms, such as tremor and bradykinesia, is poised to revolutionize clinical assessment and personalized treatment [10,11]. However, the development of wearable sensors that can reliably capture these subtle neural features while maintaining long-term skin compatibility presents a significant challenge. Conventional flexible devices often encounter fundamental limitations in achieving stable integration with tissue, as trade-offs between mechanical compliance, interfacial adhesion, and signal fidelity frequently compromise their performance in dynamic real-world environments [[12], [13], [14]].

Hydrogel materials, characterized by their unique bio-tissue-like properties, exhibit broad application prospects in the field of flexible electronics [[15], [16], [17]]. These highly water-containing materials, composed of three-dimensional crosslinked networks, not only accurately simulate the mechanical behavior and interfacial properties of biological tissues but also enable the integration of various functionalities such as electrical conductivity, sensing, and antimicrobial properties through molecular design [[18], [19], [20]]. In contrast to traditional rigid electronic materials, hydrogels can achieve mechanical properties that match those of biological tissues while maintaining excellent elasticity through adjustable cross-linking density. Furthermore, the incorporation of dynamically reversible functional group interaction networks endows these materials with self-healing capabilities and environmental adaptability [[21], [22], [23]]. However, single-component hydrogel systems exhibit significant deficiencies in achieving complex functional integration, as their performance is often constrained by the material's structural singularity. This limitation makes it challenging to simultaneously satisfy the multiple requirements of flexible electronic devices regarding mechanical properties, electrical conductivity, interfacial stability, and environmental tolerance [[24], [25], [26]].

The common substrates of hydrogels primarily include polyvinyl alcohol, polyacrylic acid, polyacrylamide, and gelatin. Among these synthetic polymers, polyacrylic acid exhibits a distinct advantage due to the abundance of carboxyl functional groups present in its molecular chain. These reactive groups facilitate the formation of reversible physical cross-linking networks and allow for the introduction of various functional properties through chemical modification [[27], [28], [29]]. Concurrently, certain natural materials play an indispensable role in hydrogel construction owing to their inherent biocompatibility and environmentally friendly characteristics, such as sodium alginate, cellulose, chitosan, and gelatin. Although natural polymeric materials demonstrate excellent biocompatibility and environmental sustainability, their intrinsic molecular structures often limit their performance within hydrogel systems. Unmodified natural materials typically suffer from insufficient cross-linking sites, limited mechanical strength, and a lack of multifunctionality, hindering their ability to meet the integration requirements of advanced hydrogels. By employing molecular modification and functionalization techniques, the processability and functionality of these materials can be significantly enhanced while preserving their biocompatibility advantages. For instance, the incorporation of aldehyde groups into the cellulose molecular chain can generate dynamic covalent cross-linking sites, endowing hydrogels with self-repairing capabilities [[30], [31], [32]]. Additionally, the methacrylation modification of gelatin facilitates the creation of various controllable cross-links, enabling precise regulation of the internal structure of hydrogels. This targeted molecular engineering approach not only addresses the intrinsic limitations of natural materials but also allows them to synergize with synthetic polymers. Dai et al. developed a dual-network hydrogel dressing utilizing dialdehyde cellulose, which forms a dynamic covalent network through the Schiff base reaction between the aldehyde group of dialdehyde cellulose and the amino group of carboxymethyl chitosan (CMCS) [33]. This approach synergistically enhances mechanical properties via the polyacrylamide covalent network. The dialdehyde cellulose facilitates self-repair by providing dynamic cross-linking sites, while simultaneously enhancing tissue adhesion with CMCS and establishing an energy dissipation mechanism that improves toughness. The hydrogel exhibits excellent mechanical strength, skin adhesion, and biocompatibility [34]. Although purely ionic conductive hydrogels possess high conductivity, they often exhibit unstable properties due to ionic migration, which can adversely affect adhesion characteristics. More critically, these monofunctional hydrogels fail to achieve a synergistic integration of sensing, self-healing, antibacterial, and other functionalities. While high water content networks mimic the softness of biological tissues, they also create a conducive environment for bacterial growth. Moreover, the self-healing capability conferred by dynamic bonding may lead to the disruption and reconstruction of the conductive network, resulting in signal drift [35,36]. This monofunctionality significantly restricts the practical application of hydrogel sensors in long-term wearable monitoring. As a naturally derived multifunctional polymer, sodium lignosulfonate contains a sulfonic acid group in its molecular structure that generates stable ion-conducting channels through dissociation, effectively enhancing the conductive stability of hydrogels and addressing the performance fluctuations typically encountered in traditional ionic hydrogels due to ion migration. Additionally, the aromatic ring structure promotes electron delocalization, forming a unique ion-electron synergistic conduction mechanism. This conductive property is seamlessly integrated with other functionalities at the molecular level; the phenolic hydroxyl group not only enhances tissue adhesion through multiple hydrogen bonds and π-π interactions but also, due to its redox activity, imparts self-repairing and antimicrobial properties to the material.

In this study, we propose a novel strategy for the design of multifunctional hydrogels based on dynamic bonding synergies, which aims to achieve an organic unity of adhesion, antimicrobial properties, and electrical conductivity. The core construction principle of this hydrogel system is rooted in a multifunctional network structure that leverages the synergistic effects of multiple components, including polyacrylic acid (PAA), dialdehyde carboxymethylcellulose (OCMC), methacrylated gelatin (GelMA), and methacrylated lignosulfonate (MLS). Specifically, the aldehyde groups of OCMC form dynamic imine bonds with the amino groups of GelMA, thereby imparting self-repairing capabilities to the network. Concurrently, the carboxyl groups of PAA and the sulfonic acid groups of MLS establish ionic crosslinking points through electrostatic interactions, while the introduced metal ions form coordination bonds with the electron-donating groups on the polymer chains, functioning as energy dissipation centers. This multimodal dynamic cross-linking design endows the hydrogel with exceptional mechanical adaptability. The reactive groups present in the MLS molecule endow the material with broad-spectrum antimicrobial properties. A mixed ion-electronic conduction mechanism, facilitated by free ions from PAA and MLS, ensures stable electrical signal transmission. Exceptional adhesion is achieved through surface-enriched catechol groups, which can form multiple interactions with skin surface proteins, resulting in strong adhesion. Notably, all functional components are immobilized within the polymer network via covalent bonding, preventing the loss of functional molecules and ensuring long-term stability. This multi-component synergistic design overcomes the limitations of traditional hydrogels, which often exhibit only single performance characteristics. Through precise molecular-level regulation, it achieves an optimal balance of adhesion, antimicrobial activity, and electrical conductivity, without relying on asymmetric structures. These properties enable the materials to function as flexible strain sensors for monitoring human physiological signals and Parkinson's symptoms, thereby providing a novel material platform for the development of next-generation smart medical sensors.

## 2 Results and discussions

### 2.1 Design and characterization of AGOM hydrogel

**Fig. 1:**
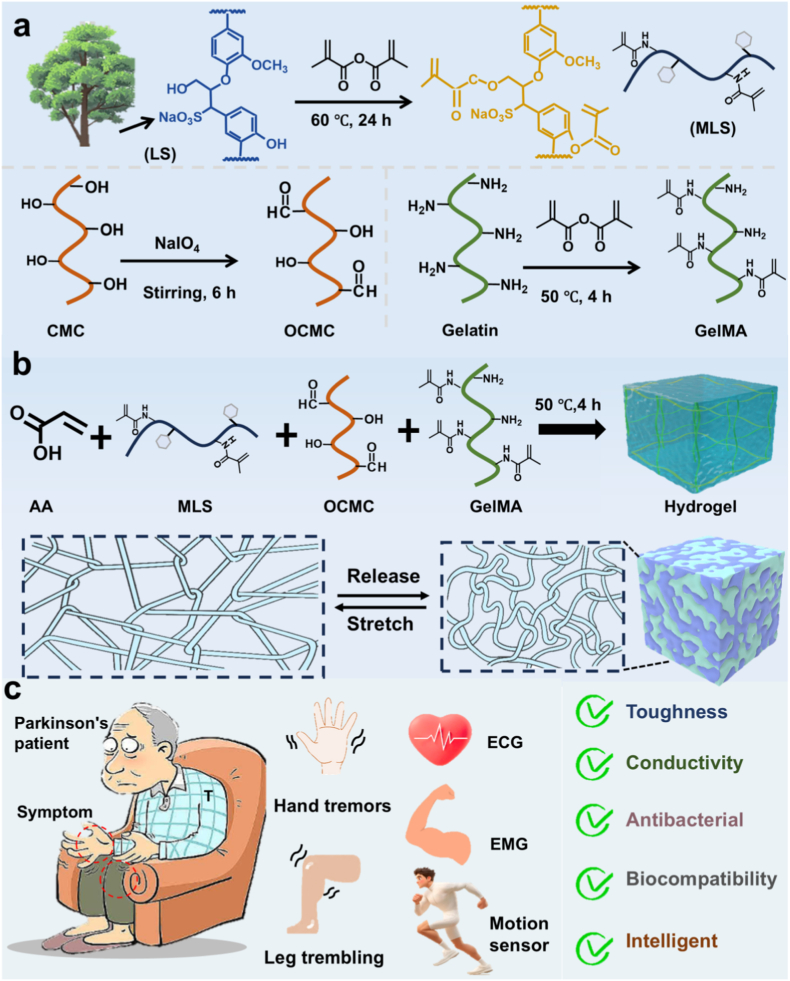
(a) Synthesis diagram of the corresponding components of the hydrogel. (b) Preparation of hydrogels. (c) Applications and properties.

**Fig. 2:**
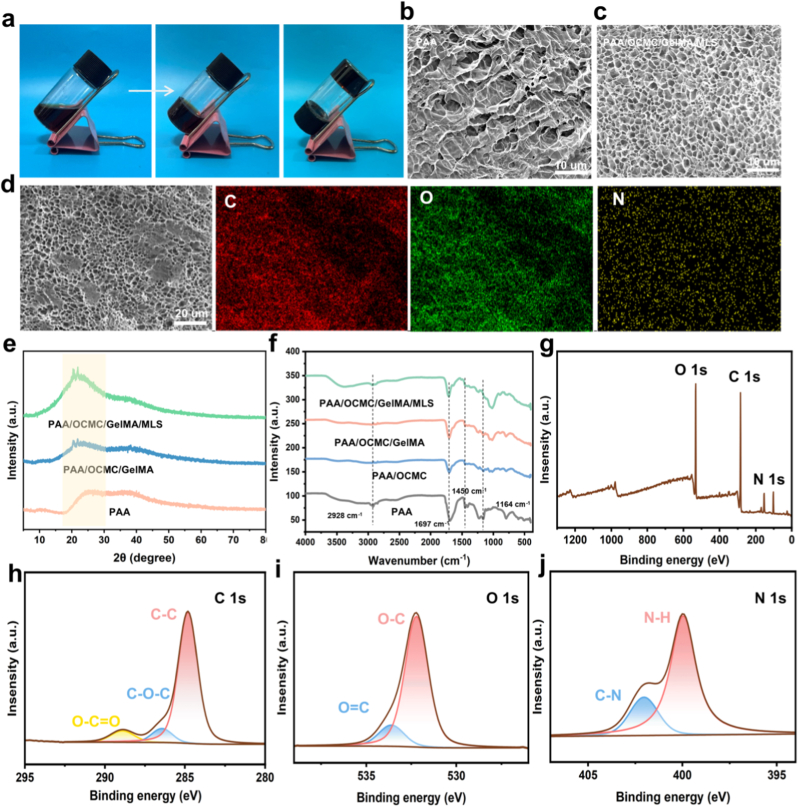
Characterization of AGOM hydrogel. (a) Inverted view of the vial showing the state of hydrogel formation. (b) SEM comparison of PAA hydrogel and AGOM hydrogel. (c) Elemental (C, N and O) distribution of AGOM hydrogel. (d) XRD spectra of different hydrogel samples. (e) FT-IR spectra of different hydrogel samples. (f–i) XPS spectra of AGOM hydrogels with C, N and O elements.

In this study, we propose a multicomponent synergistic thermal polymerization strategy (Fig. 1a–c) to successfully construct functionalized hydrogels with a gradient network structure via the in situ thermal polymerization reaction of acrylic acid (AA), dialdehyde carboxymethylcellulose (OCMC), sodium methacrylated lignosulfonate (MLS), and methacrylated gelatin (GelMA). This strategy employs dynamic covalent cross-linking and multiple non-covalent interactions among the components to achieve a synergistic enhancement of material properties. The aldehyde group of OCMC forms a reversible imine bond with GelMA, imparting self-repairing properties to the material. Furthermore, the sulfonic acid and phenolic hydroxyl groups of MLS provide adhesion sites and conductive pathways, while the bioactive skeleton of GelMA optimizes interfacial compatibility. This multicomponent synergy enables the hydrogel to combine excellent interfacial adhesion and stable conductivity while maintaining high elasticity, making it an ideal interfacial material for wearable bioelectronic devices. As shown in Fig. 2a, the hydrogels were polymerized in vials by a simple one-pot method of heating to form uniform hydrogels. As shown in Fig. 2b and c, SEM images demonstrated that the pure PAA hydrogels exhibited loose and inhomogeneous pore sizes, however, upon doping with OCMC, GelMA and MLS, the hydrogels formed a denser pore structure, and dense pore sizes were clearly observed in the AGOM hydrogels.The EDS mapping images verified the homogeneous distribution of the C, O, and S elements within the hydrogels, confirming that these components were uniformly distributed (Fig. 2d). XRD analysis of the poly (acrylic acid) hydrogels showed a distinct diffraction peak at the center of approximately 2θ = 24°, with no other sharp diffraction peaks observable, confirming the amorphous nature of the material. The incorporation of multiple components (OCMC, GelMA, and MLS) combined to inhibit the formation of crystalline domains. A homogeneous network structure typical of highly hydrated polymer systems was formed (Fig. 2e).

Fig. 2f shows the FTIR spectra of the four hydrogels. The characteristic peaks at 2928 cm^−1^, 1697 cm^−1^, 1450 cm^−1^ and 1164 cm^−1^ correspond to the tensile vibration of C-H and carboxyl C=O, hydroxyl O-H and C-O, respectively. It is noteworthy that the PAA hydrogel has a peak C-O tensile vibration at 1164 cm^−1^. However, in PAA/OCMC/GelMA/MLS hydrogels, the peak of tensile vibration was found to disappear. This may be due to the formation of a new hydrogen bonding network within the network, evidenced by the introduction of its new substance, which changes the local chemical environment of the C-O bonds and removes the peak. In addition, the electrostatic interactions between the sulfonic acid groups and PAA may further modify the PAA crosslinked network and affect its C-O vibration mode.

Fig. 2g presents the X-ray photoelectron spectroscopy (XPS) spectra of the AGOM hydrogel along with the deconvoluted high-resolution spectra of C 1s, O 1s, and N 1s regions. In the C 1s spectrum, three characteristic peaks were identified through peak fitting: 284.09 eV (C-C), 286.4 eV (C-O), and 289.1 eV (O=C-O), confirming the successful incorporation of all organic components. The N 1s spectrum exhibited two predominant peaks at 400.2 eV (N-H) and 402.1 eV (C-N), demonstrating the preserved protein functionality. The O 1s region revealed two distinct components at 532.2 eV (C=O) and 533.5 eV (C-O), indicating effective cross-linking formation. These characteristic peaks provide further evidence for the synergistic interactions among MLS, OCMC, and GelMA within the hydrogel network.

### 2.2 Mechanical properties of AGOM hydrogels

**Fig. 3:**
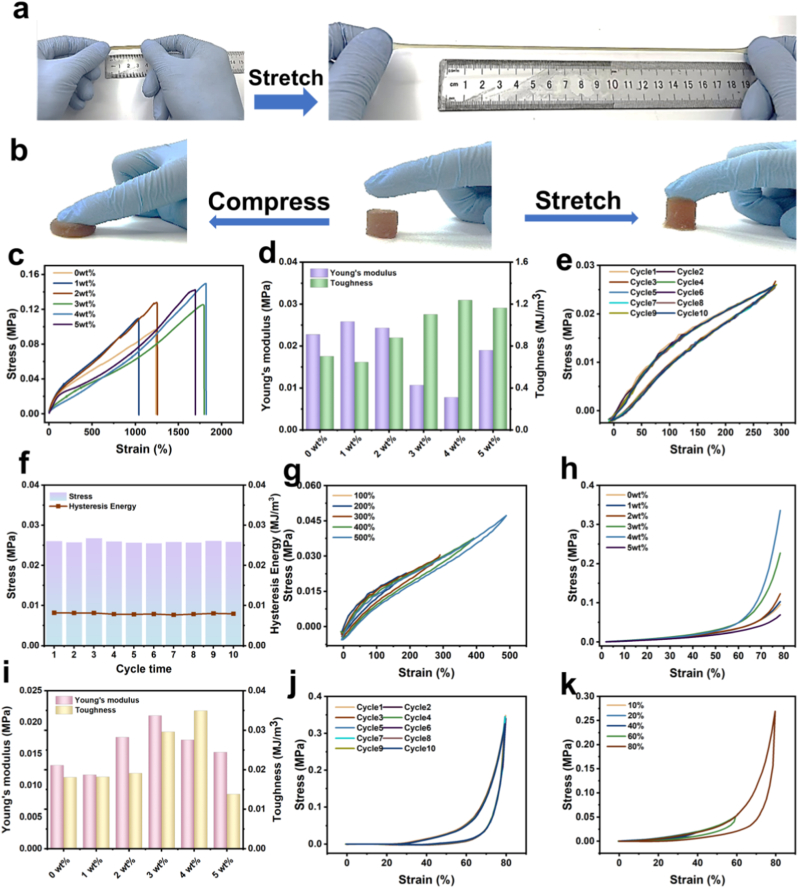
Mechanical Properties of AGOM hydrogels (a) The hydrogel is capable of being stretched to more than seven times its original length. (b) The hydrogel quickly returns to its original height after being pressed. (c) The tensile stress-strain curves of hydrogels with varying MLS contents are presented. (d) The corresponding Young's modulus and toughness are illustrated. (e) Tensile stress-strain curves of AGOM_4_ hydrogels subjected to ten consecutive tensile cycles are shown. (f) The tensile strength and dissipated energy for various tensile cycles at a fixed strain are discussed. (g) Tensile cycling curves for different displacements are provided. (h) Compressive stress-strain curves for hydrogels with different MLS contents are depicted. (i) The compression Young's modulus and toughness are analyzed. (j) Curves representing AGOM_4_ hydrogels subjected to ten consecutive compression cycles are included. (k) Compression cycle curves for different displacements are presented.

Excellent mechanical properties are crucial for the practical applications of hydrogels [[37], [38], [39], [40]]. As illustrated in Fig. 3a and b, AGOM hydrogels demonstrate remarkable mechanical properties, capable of stretching to more than seven times their original length. Furthermore, these hydrogels exhibit rapid recovery after compression, returning to their initial height while maintaining structural stability under load. We specifically evaluated a range of mechanical properties of the hydrogels. The typical stress-strain curves are presented in Fig. 3c, which shows that as the MLS content increases, the mechanical properties correspondingly improve. At an MLS content of 4 wt%, the tensile strength and elongation at break reached 0.146 MPa and 1820 %, respectively. Similarly, Young's modulus and toughness significantly increased to 78.2 kPa and 1.26 MJ/m^3^, respectively (Fig. 3d). This enhancement is attributed to the increased hydrogen bonding and polymerization within the hydrogel network as the MLS content rises, thereby improving the mechanical properties. However, when the MLS content exceeds a certain threshold, the excessive density of hydrogen bonding can lead to a decline in the mechanical properties of the hydrogels.

Subsequently, we conducted a series of cyclic loading tests to evaluate the fatigue resistance of AGOM hydrogels. The cyclic curves at a fixed tensile strain of 300 % are presented in Fig. 3e. The ten consecutive tensile cycles nearly overlapped, and the corresponding tensile strengths and dissipated energies are illustrated in Fig. 3f, revealing a low hysteresis. As the strain increases from 100 % to 500 %, the area enclosed by the corresponding cycle curves also increases, indicating a rise in energy dissipation (Fig. 3g). This phenomenon can be attributed to the increased energy available to the hydrogel's internal network, enabling it to resist external damage during tensile deformation.

In addition to possessing excellent tensile strength, compressive strength is also crucial for practical applications. As illustrated in Fig. 3h, the hydrogel containing 4 wt% MLS exhibits the highest compressive properties, with a maximum compressive strength of 0.35 MPa, which is 3.68 times greater than that of the hydrogel with 0 wt% MLS. Similarly, the Young's modulus and toughness of the hydrogel reached 0.017 MPa and 0.0349 MJ/m^3^, respectively (Fig. 3i). Fig. 3j presents the stress-strain curves of the hydrogels subjected to ten consecutive tensile strain cycles. The results indicate that the cyclic curves nearly overlap, and the stress remains relatively constant throughout the ten cycles. Furthermore, the maximum stress and hysteresis energy of the hydrogel remain nearly constant over ten compression cycles, demonstrating its excellent fatigue resistance. When exposed to mechanical forces, the stresses are more evenly distributed within this network, thereby preventing stress concentration and enhancing both the tensile and compressive strength of the hydrogel. Fig. 3k shows that the hydrogel retains exceptional elasticity and durability during compression cycles at varying displacements. This is attributed to the reversible cross-linking bonds formed within the hydrogel network, which, under the synergistic influence of multiple components, can withstand external forces and effectively confer elasticity and fatigue resistance to the hydrogel.

### 2.3 Adhesion properties of AGOM hydrogels

**Fig. 4:**
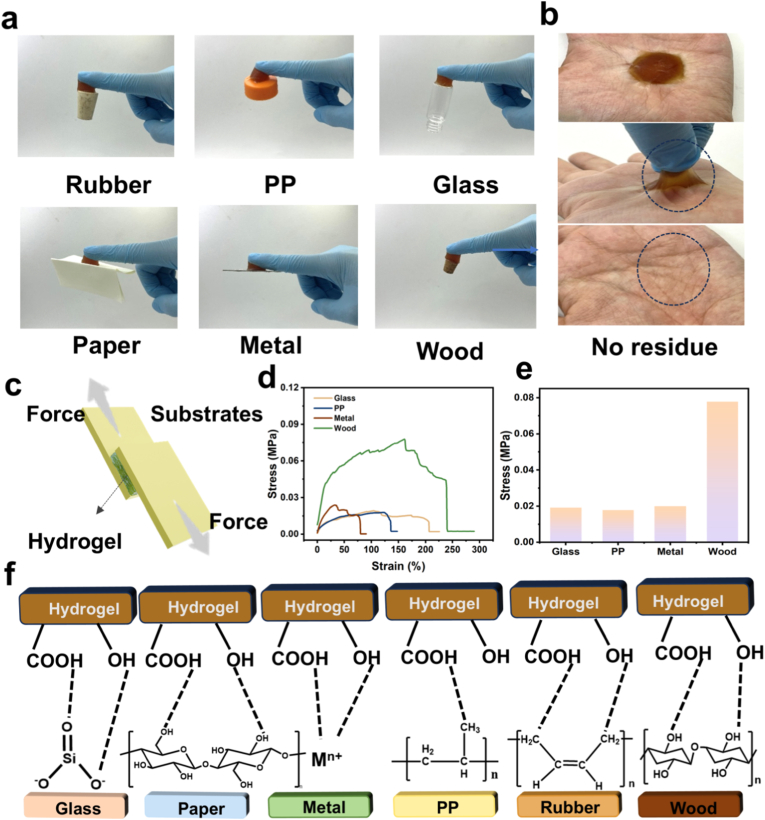
The adhesion properties of AGOM hydrogels are examined through several key aspects. (a) The adhesion of the hydrogel to various organic and inorganic materials is analyzed. (b) The hydrogel demonstrates residue-free adhesion on the surface of a hand. (c) A schematic representation of the lap-shear adhesion test is provided. (d) Adhesion curves illustrating the hydrogel's interaction with different materials are presented. (e) The adhesion strength is quantified. (f) A diagram depicting the adhesion mechanism of the hydrogel is included.

AGOM hydrogels exhibit excellent adhesion properties, forming tight and stable bonds with a wide range of material surfaces [[41], [42], [43]]. As illustrated in Fig. 4a, the hydrogel can adaptively adhere to substrates of varying shapes and materials, including rigid materials such as plastic, metal, and glass, as well as flexible surfaces like rubber, wood, and pigskin. Notably, it demonstrates exceptional self-adhesion capabilities. Particularly remarkable is its strong bonding with human skin (Fig. 4b), which maintains firm adhesion during dynamic movements and leaves no residue upon removal, thereby avoiding skin irritation commonly associated with conventional adhesives. This outstanding adhesion property arises from its unique network structure: AA, MLS, and OCMC, along with GelMA, form a three-dimensional cross-linking framework through high-density hydrogen bonding. This structure, combined with the synergistic effects of hydrophobic interactions and polymer chain entanglement, effectively inhibits water migration, resulting in strong adhesion to various substrates. This multi-mechanism synergistic adhesion design not only broadens the application scope of hydrogels in wearable sensors but also significantly enhances signal acquisition accuracy by reducing interfacial contact resistance.

As illustrated in Fig. 4c, the adhesion properties of AGOM hydrogels were assessed through standard tensile tests, wherein the hydrogels were positioned between test substrates and subjected to pressure to ensure optimal contact. The results indicated that the AGOM hydrogel demonstrated exceptional interfacial bonding with various substrates. As depicted in Fig. 4d and e, the bond strength curves reveal that the maximum adhesion strength of the hydrogel can attain 77.8 kPa, a value that substantiates its strong adhesive capability on diverse material surfaces. This remarkable adhesion performance is primarily attributed to the incorporation of MLS and its synergistic interaction with other components. Specifically, the carboxyl group of PAA, the catechol and sulfonic acid groups of MLS, and the abundant hydroxyl groups of OCMC collaboratively enhance the interfacial bonding strength between the hydrogel and substrate material through various intermolecular forces, including hydrogen bonding, electrostatic interactions, and metal liganding (Fig. 4f). This multi-mechanism synergistic adhesion strategy not only provides AGOM hydrogels with exceptional universal adhesion capability but also allows them to adhere firmly to complex surfaces, such as skin, while facilitating easy peeling and residue-free removal. Given its outstanding adhesion performance, the AGOM hydrogel presents significant application potential in wearable electronics, biomedical sensing, and flexible devices. Its stable interfacial bonding ability can effectively minimize signal interference and enhance the monitoring accuracy of sensors, offering an ideal adhesive material solution for the next generation of high-performance flexible electronic devices.

### 2.4 Sensing properties of AGOM hydrogels

**Fig. 5:**
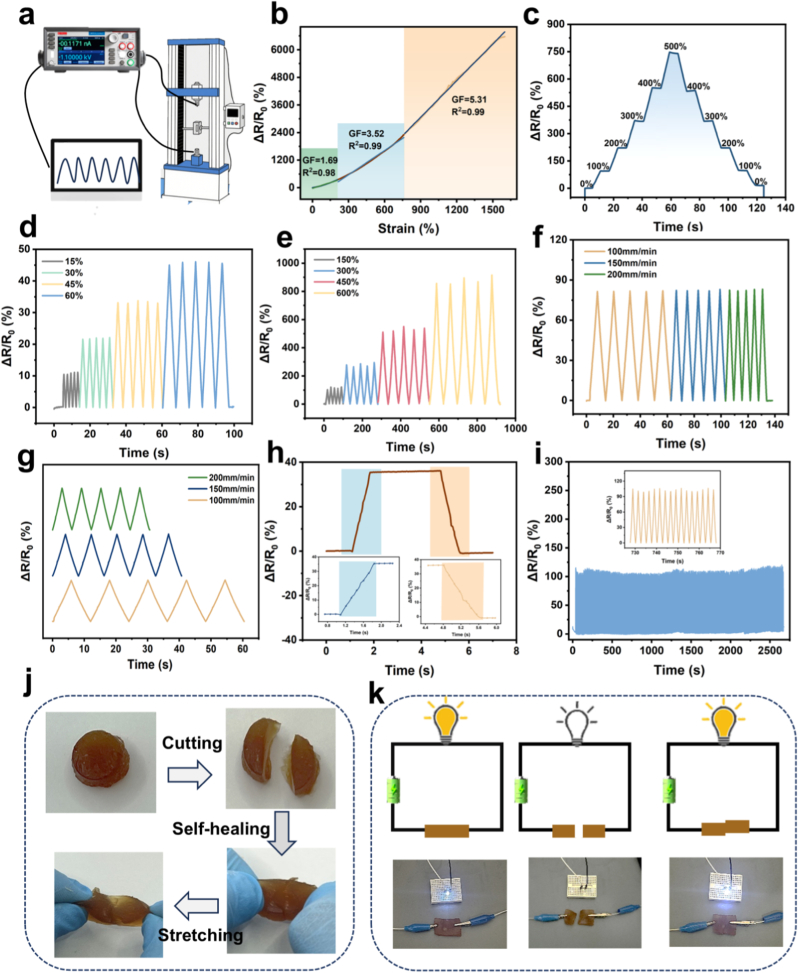
Sensing performance of AGOM hydrogel. (a) Schematic of the lap device for sensing performance testing. (b) Relative resistance change of AGOM hydrogel during stretching from 0 % to 1500 %. (c) Rate of change of resistance at different strains. (d) Change in resistivity for small strain stretching. (e) Change in tensile resistivity at large strains. (f–g) Resistivity change at different stretching speeds. (h) Change in response to microstrain. (i) Relative resistivity change for 1000 consecutive loading-unloading cycles at 50 % strain and the corresponding magnified plot of cyclic response. (j) Self-healing test of hydrogel in cut contact. (k) Hydrogel attached to a line with an LED bulb.

As illustrated in Fig. 5a, AGOM hydrogels exhibit promising characteristics as flexible strain sensors capable of monitoring resistance-based signal variations, attributed to their remarkable flexibility and adhesion properties. In particular, we assessed the strain sensing performance of AGOM hydrogel sensors. The strain sensitivity of hydrogels is commonly evaluated by the change in relative resistance (*ΔR/R*_*0*_) across different strains. The gauge factor (GF) serves as a critical metric for sensor performance [[44], [45], [46], [47]]. As depicted in Fig. 5b, the relative resistance of the AGOM hydrogel demonstrates a linear response across three distinct regions of tensile strain: 0 %–250 %, 250 %–750 %, and 750 %–1500 %. The calculated GF values are 1.69, 3.52, and 5.31, respectively, indicating a strong responsiveness across a broad spectrum of strains.

The AGOM hydrogel demonstrates exceptional dynamic sensing performance characterized by remarkable sensitivity and stability across a broad strain range. As illustrated in Fig. 5c, the relative resistance change of the sensor shows a highly stable stepwise increase during the progressive strain test, ranging from 0 % to 500 %, and exhibits a strong correlation with the strain level. Notably, the hydrogel sensor possesses an extensive strain detection range, accurately responding to small strains between 15 % and 60 %, while also maintaining stable electrical signal output under significant deformations from 150 % to 600 % (see Fig. 5d and e). In the small strain region, the resistance change exhibits a highly linear relationship with strain, indicating excellent microstrain detection capabilities. Furthermore, under large strain conditions, the sensor continues to exhibit significant linear response characteristics, rendering it suitable for monitoring deformations of varying amplitudes.

To further verify the dynamic performance, we tested the response characteristics under varying stretching rates (50–150 mm/min) (Fig. 5f and g). The experimental results indicate that the resistance signals can accurately track deformation changes in real time and maintain stability across different strain rates, thereby fulfilling the stringent requirements of wearable devices for dynamic signal acquisition. As illustrated in Fig. 5h, the sensor demonstrates rapid responsiveness even at small strains. Notably, the resistance response curves of the sensor remain highly consistent over 1000 cyclic tensile tests (Fig. 5i), with the amplified view revealing that the signals from each cycle are nearly completely overlapped, confirming its exceptional long-term operational stability. This combination of stable cycling performance and rapid response characteristics positions the AGOM hydrogel as an ideal sensing material for monitoring human joint movements, muscle contractions, and other physiological activities. AGOM hydrogels exhibit exceptional self-healing performance, a characteristic crucial for prolonging device lifespan and minimizing maintenance costs. As illustrated in Fig. 5j and k, when the hydrogel is severed, the sections can self-heal at room temperature with merely 1 min of contact. The repaired samples can endure substantial tensile stress without fracturing, thereby demonstrating their effective self-healing capability. After 6 h of natural bonding at room temperature, the mechanical strength and elongation at break still reached 65.8 % and 61 % of the initial hydrogel, respectively, meeting the mechanical requirements for wearable devices (Fig. S1). Notably, this self-healing behavior is evident not only in the recovery of macroscopic mechanical properties but also in the rapid restoration of electrical functions. Conductivity test experiments reveal that when the intact hydrogel circuit is interrupted, the LED indicator extinguishes immediately. However, once the fractured surfaces are reconnected, the circuit's conductivity is restored instantaneously, and the LED is relit within a short time frame. Our tests indicate that the electrical conductivity of AGOM can reach as high as 0.55 S/m (Fig. S2). This impressive conductivity, coupled with the dynamic reconfiguration and rapid migration capabilities of the conductive network within the hydrogel, provides a reliable foundation for its practical application in the field of flexible electronics.

### 2.5 Human motion monitoring sensors and morse code transmission

**Fig. 6:**
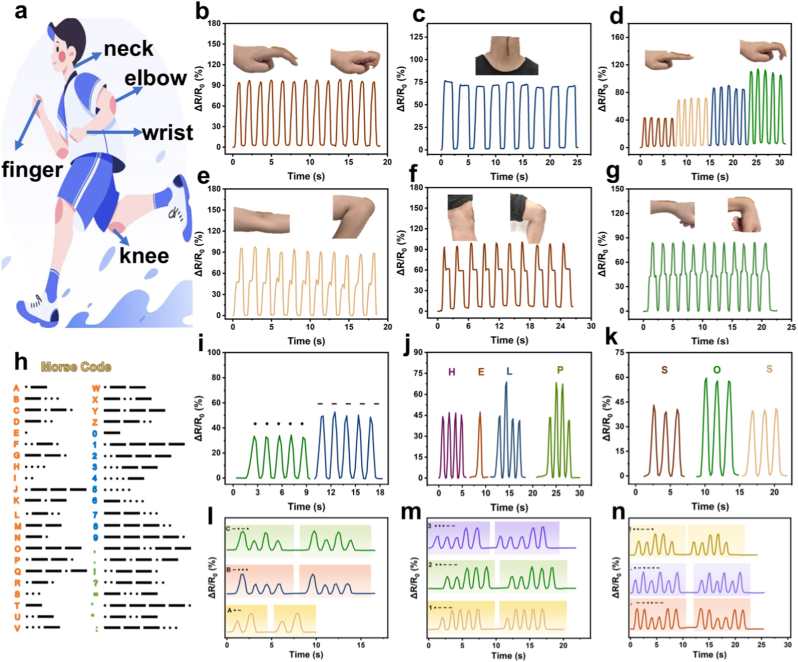
Applications of AGOM hydrogel sensors in human motion monitoring and encrypted Morse code communication. (a) Schematic diagram showing the placement of hydrogel sensors on various joints of the human body for motion monitoring. Real-time relative resistance changes of the AGOM hydrogel sensor for different human movements: (b) Finger flexion and extension. (c) Swallowing action. (d) Finger bending at different angles. (e) Elbow joint flexion. (f) Knee joint flexion. (g) Wrist rotation. (h) Schematic of Morse code encoding principle: Short signal (.) and long signal (−) correspond to little finger bend amplitude and thumb bend amplitude, respectively. (i) Stable resistance response signal corresponding to repeated Morse code input. Demonstration of successful encrypted information transmission by the sensor system: (j) Distress message “HELP”. (k) Emergency signal “SOS”. (l) Letters (A, B, C). (m) Numbers (1, 2, 3). (n) Symbols (! .).

AGOM hydrogel sensors have demonstrated exceptional performance in human motion monitoring due to their superior electromechanical response characteristics. By integrating these sensors into various motion-critical areas of the human body, the system facilitates precise monitoring of complex physiological activities (Fig. 6a–g). Further multi-joint motion tests reveal that the sensor can accurately distinguish between the motion characteristics of different joints, generating significantly different resistive response signals under complex motion patterns, such as elbow flexion/extension, wrist rotation, knee movement, finger bending at various angles, and multi-directional neck movement. These attributes render the sensors valuable in clinical motor function assessment, rehabilitation medicine monitoring, and intelligent human-computer interaction, thereby providing a reliable means for biomechanical data acquisition in precision medicine.

AGOM hydrogel sensors exhibit significant potential for multifunctional applications, facilitating both information coding and decoding functions alongside human motion monitoring. Leveraging their sensitive strain-resistance response characteristics, we employed the internationally recognized Morse coding principle (Fig. 6h) to differentiate coding units by recognizing varying amplitudes of finger flexion movements. In the experiment, the hydrogel sensor was affixed to the subject's knuckle, and the coding rules were established based on the correlation between the degree of joint flexion and the corresponding change in resistance: a small-amplitude flexion is detected as a short message number '.', while a larger flexion corresponds to the long signal '-'. The sensor accurately distinguishes between two different amplitude bending motions and outputs distinct electrical signal differences. Furthermore, in continuous repeated tests (Fig. 6i), the sensor demonstrates stable signal output performance, and the electrical signal characteristics of different encoding units remain highly consistent, thereby verifying its reliability in message encryption transmission applications. This information encoding method is based on biomechanical actions. It offers a novel technical approach for human-computer interaction in wearable devices. Additionally, it broadens the application prospects of flexible electronics in information security.

To verify the feasibility of message transmission, the results are presented in Fig. 6j–n. The sensor successfully transmits letters (A, B, C), numbers (1, 2, 3), and symbols (! and. And). In further functional tests, the system successfully encoded the transmission of complete words, including the emergency signal “SOS” and the help message “HELP.” These experimental results confirm that the sensor system is capable of multilevel information processing. Its stable signal output characteristics and accurate encoding recognition performance open new avenues for the application of flexible electronic devices in the field of encrypted communication.

### 2.6 Applications in human bioelectronics

**Fig. 7:**
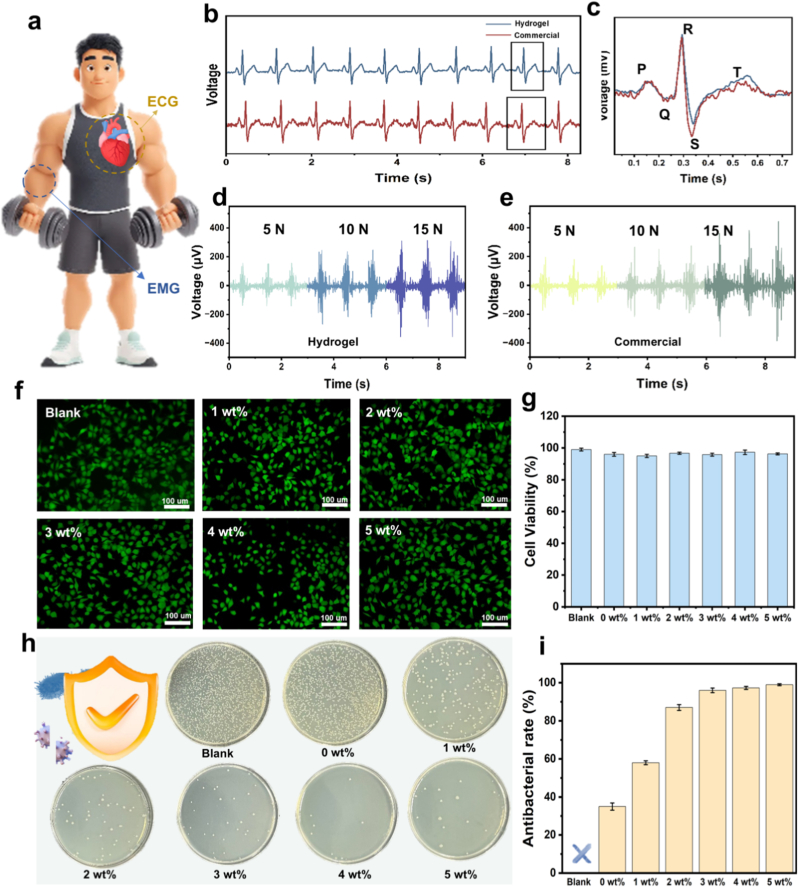
(a) Schematic of human ECG and EMG. (b) ECG measured with commercial and gel electrodes. (c) Localized magnified view of ECG measured with commercial and gel electrodes. (d–e) EMG measured with commercial and gel electrodes. (f) Cytocompatibility test, stained image of cells after co-culture with hydrogel material. (g) Cell survival. (h) Antimicrobial assay. (i) Bacterial inhibition rate.

AGOM hydrogels are anticipated to be utilized as physiological signal detection electrodes due to their outstanding adhesion properties and electrical conductivity. Electrocardiography (ECG) captures the electrical activity generated during each cardiac cycle from the body surface, serving as a crucial guide for health assessment [[48], [49], [50]]. Nevertheless, achieving stable and accurate ECG monitoring remains a significant challenge. We recorded ECG signals using both commercially available electrodes and AGOM hydrogel electrodes (Fig. 7a). During the monitoring process, the signals obtained from the hydrogel electrodes and the commercially available electrodes were nearly identical, with the AGOM hydrogel demonstrating superior signal stability compared to the commercially available gel electrodes (Fig. 7b and c). In contrast, the AGOM hydrogel is characterized by its high skin adhesion and electrical conductivity, which provide excellent ionic conductivity, thereby facilitating the formation of stable conductive channels between the electrodes and the skin to ensure effective and stable signal transmission. The AGOM hydrogel can be seamlessly adhered to the skin's surface, allowing for a tight fit that enhances signal acquisition quality. This design prevents signal interruptions caused by gel detachment. Additionally, the hydrogel's high sensitivity guarantees the faithful transmission of weak signals.

The AGOM hydrogel demonstrates significant advantages in the field of physiological electrical signal detection due to its excellent skin adhesion and conductive properties. As an electrode material for electrocardiogram (ECG) monitoring, this hydrogel accurately records changes in body surface potentials generated by the electrical activity of the heart. Experimental studies comparing the performance of conventional commercial electrodes with AGOM hydrogel electrodes reveal that the waveforms of ECG signals obtained from both types of electrodes are highly consistent, while the hydrogel electrodes exhibit superior signal stability. Furthermore, AGOM hydrogel possesses excellent biocompatibility and conductivity, making it an ideal medium for the acquisition of bioelectrical signals. Its outstanding surface adhesion properties ensure a tight fit with skin tissue, forming a low-impedance contact interface. Additionally, the three-dimensional conductive network structure within the hydrogel provides an efficient channel for ion transmission, thereby ensuring the complete transmission of electrical signals.

To further evaluate the performance advantages of AGOM hydrogel electrodes, the researchers conducted comparison experiments on EMG acquisition. As illustrated in Fig. 7d and e, volunteer testing revealed that the hydrogel electrode exhibited superior EMG signal acquisition performance compared to traditional commercial electrodes. The experiments were designed to record EMG signals across varying grip force levels, and the results indicated that the hydrogel electrodes maintained stable signal output quality under all test conditions. This performance advantage is primarily attributed to the low impedance interface established between the material and the skin tissue, alongside its exceptional mechanical adaptability, which effectively mitigates the generation of motion artifacts. The findings of this study affirm the potential application of AGOM hydrogel as a high-performance bioelectrode material, particularly in EMG signal acquisition scenarios that necessitate long-term stable monitoring. The reliable signal monitoring offers valuable detection data for clinical diagnosis. Collectively, these attributes underscore the significant application value of AGOM hydrogel in the realm of wearable medical monitoring. To evaluate the biosafety of AGOM hydrogel, this study employed the L929 cell model for in vitro biocompatibility testing, utilizing the live/dead cell staining method (Fig. 7f and g). The cells co-cultured with the hydrogel maintained a typical shuttle-shaped morphology and exhibited a survival rate exceeding 98 %, significantly surpassing the biocompatibility standards. This confirms the material's excellent cytocompatibility, suggesting that hydrogel-based wearable sensors can be applied in various contexts. Furthermore, the antimicrobial properties of wearable sensors are critical for applications involving prolonged skin contact. In this study, we assessed the inhibitory effect of AGOM hydrogel on two common pathogenic bacteria, *E.coli* and *S.aureus*, through standard antimicrobial experiments (Fig. 7h and i). The experimental data revealed that the hydrogels displayed enhanced antimicrobial activity with increasing MLS content, demonstrating significant growth inhibition against both strains. This improvement is primarily attributed to the interference of phenolic hydroxyl groups and other reactive functional groups with bacterial metabolism, leading to oxidative damage. These findings indicate that AGOM hydrogels possess excellent antimicrobial properties, effectively enhancing safety during wear. They fully meet the biosafety requirements for wearable biosensors intended for long-term skin contact applications.

### 2.7 Machine learning for Parkinson's gait recognition

In this study, a wearable intelligent monitoring system based on hydrogel sensors was developed to accurately identify and classify motor symptoms in Parkinson's patients [[51], [52], [53], [54]]. Owing to the hydrogel's excellent adhesion, sensitivity, and antimicrobial properties, it is particularly well-suited for continuous and non-invasive health monitoring applications. The system targets two of the most common motor symptoms associated with Parkinson's disease: hand tremors and leg tremors, with the goal of providing real-time symptom detection and facilitating intelligent healthcare interventions.

**Fig. 8:**
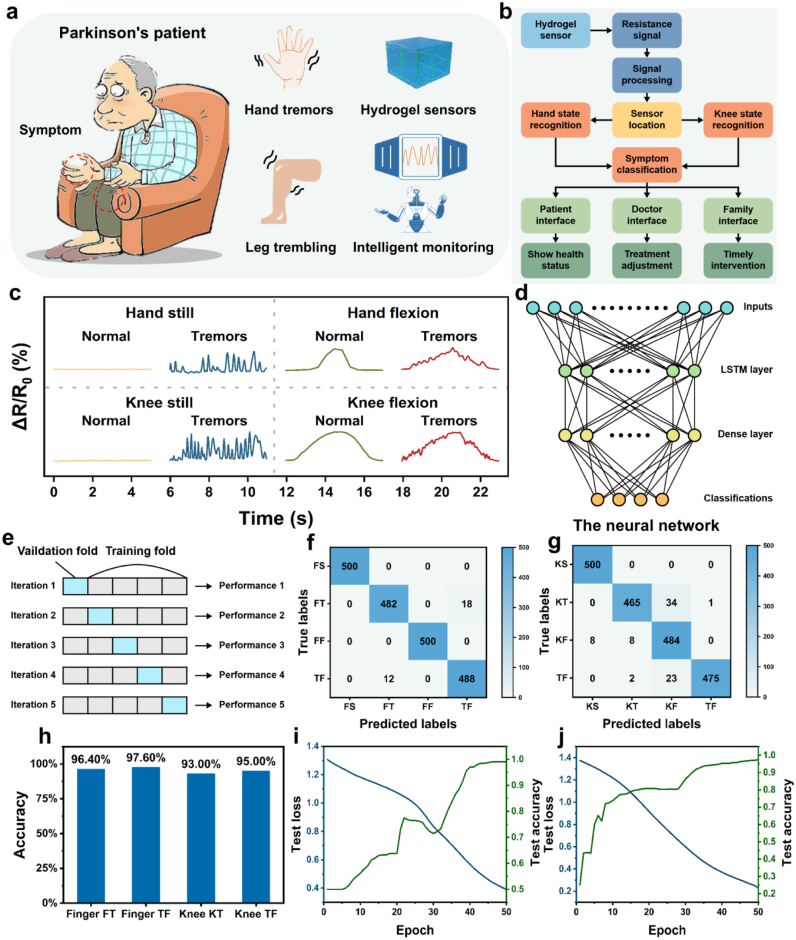
Overview of the intelligent monitoring system for Parkinson's patients using hydrogel-based sensors. (a) Schematic of the hydrogel sensor designed to detect motor symptoms. (b) Workflow of the complete monitoring system with interfaces for the patient, doctor, and family. (c) Real-time resistance monitoring across eight different body postures. (d) Architecture of the neural network used for symptom recognition based on resistance signals from the hydrogel sensors. (e) Illustration of the 5-fold cross-validation method. (f–g) Confusion matrices for hand (f) and knee (g) symptom recognition, with four classes: Still (FS/KS), Tremors (FT/KT), Flexion (FF/KF), and Tremors during Flexion (TF). (h) Accuracy comparison for tremor-related symptoms (FT, KT, TF). (i–j) Test loss and accuracy during training of the hand (i) and knee (j) recognition models on one representative fold.

As illustrated in Fig. 8a, the system monitors the motor status of Parkinson's patients by detecting resistance changes induced by movement-related deformation of the hydrogel sensors attached to key anatomical sites. Fig. 8b outlines the complete data processing workflow, from resistance signal acquisition through hydrogel sensors to signal processing, localization, classification, and finally, output to patient, physician, and caregiver interfaces. This workflow enables timely symptom classification and health status feedback. The patient interface provides users with real-time health updates, the physician interface supports treatment adjustment based on symptom trends, and the family interface enables prompt intervention when abnormal conditions are detected.

Fig. 8c presents the resistance signal variation across eight distinct body postures, comprising both still and flexion states for the hand and knee, each with and without tremors. These postures correspond to specific motor symptoms, and the associated resistance signals exhibit characteristic waveforms that are used for classification. The temporal dynamics of these resistance profiles are critical for distinguishing between subtle motor abnormalities, especially in cases involving tremors during limb flexion or rest. To effectively capture the sequential nature of the resistance signals, a neural network model incorporating a long short-term memory (LSTM) layer followed by a dense classification layer was constructed, as shown in Fig. 8d. Given the high temporal dependency of the input signals, the LSTM layer enables the model to learn time-dependent features by preserving long-range dependencies. Input sequences of resistance values over fixed time intervals are continuously fed into the model, allowing for real-time prediction. Two separate models were trained for hand and knee symptoms respectively, considering the anatomical and signal pattern differences between these regions.Model training and evaluation were performed using five-fold cross-validation to ensure robust performance estimation and improved generalizability, as illustrated in Fig. 8e. Each fold consisted of an 80:20 training-testing split rotated across five iterations. The neural network was optimized with a carefully selected set of hyperparameters to enhance convergence speed and classification accuracy.

Fig. 8f and g shows the confusion matrices for symptom recognition in the hand and knee, respectively. In the hand model, the recognition of hand still and hand flexion symptoms achieved near-perfect classification, with only 18 misclassifications between hand tremors and tremors during hand flexion. The knee model, while slightly more complex due to overlapping signal characteristics, also demonstrated high performance. Knee still and knee flexion were correctly classified in the majority of cases, with some misclassifications among tremor-related classes. Specifically, tremors during knee flexion were occasionally confused with simple flexion or rest tremors, suggesting subtle overlaps in resistance signal features in dynamic motion scenarios. The overall classification accuracies of the four motor symptoms are shown in Fig. 8h. The model achieved 96.40 % accuracy for hand tremors, 97.60 % for tremors during hand flexion, 93.00 % for knee tremors, and 95.00 % for tremors during knee flexion. These results validate the model's ability to effectively differentiate between closely related symptoms and support its application in real-world Parkinson's monitoring scenarios. Fig. 8i and j depict the training progress of the hand and knee models, respectively. Both models exhibited a steady decline in test loss and consistent improvement in test accuracy with increasing epochs, demonstrating the stability and effectiveness of the learning process. The learning curves further confirm the reliability of the system for consistent long-term deployment in symptom monitoring tasks. Collectively, the results demonstrate that the hydrogel-based wearable sensor system, combined with neural network-based analysis, enables precise and real-time detection of Parkinsonian motor symptoms. By leveraging physiological resistance signals to infer symptomatic states, this system provides a promising approach for intelligent disease monitoring, supporting patients in daily life, aiding physicians in clinical decision-making, and enabling caregivers to implement timely interventions.While the developed sensor demonstrates excellent performance in physiological signal monitoring, all current validations have been conducted with healthy subjects under controlled conditions. Future studies will focus on clinical validation with specific patient populations (e.g., Parkinson's disease) and under medication scenarios to advance practical clinical applications.

## 3 Conclusion

In this study, we successfully developed a smart hydrogel sensing system based on dynamic covalent cross-linking. Through a multicomponent synergistic design of PAA/OCMC/GelMA/MLS, we achieved the synergistic optimization of mechanical properties, including mechanical strength (>0.146 MPa), toughness (>1.26 MJ/m^3^), and electrical conductivity. The hydrogel exhibits excellent biocompatibility, with a cell viability greater than 98 %, and demonstrates long-lasting antimicrobial properties, achieving bacterial inhibition rates exceeding 99 %. Furthermore, it can form a stable conformal contact interface with the skin, providing a high-precision sensing platform for monitoring motor symptoms of Parkinson's disease. The system accurately identifies characteristic signals and manifestations of motor delay. Serving as a hydrogel electrode for EMG and ECG, it exhibits stability and accuracy in signal recording. Additionally, the use of the hydrogel as a strain sensor facilitates encrypted information transmission and wearable motion monitoring. A Parkinson's numerical analysis model, constructed using machine learning algorithms, demonstrated an impressive 97.6 % accuracy in symptom classification. This research promotes the leapfrog development of flexible electronics, transitioning from single sensing to intelligent diagnostic systems. It holds significant application value in areas such as early screening for Parkinson's disease and human-computer interaction, providing an innovative example of the deep integration of smart materials and medical artificial intelligence.

## CRediT authorship contribution statement

**Siqi Ding:** Writing – original draft. **Xiao Yu:** Formal analysis. **Qi Wang:** Project administration. **Peng Luo:** Project administration. **Hua Li:** Investigation. **Zhengrui Li:** Formal analysis. **Ruhan Wang:** Conceptualization. **Hengrui Liu:** Investigation. **Yucang He:** Funding acquisition. **Jinyao Nong:** Software. **Chao Zhang:** Project administration.

## Ethical standards

All experiments in this study were conducted in compliance with the current laws and regulations of the country/region where the experiments took place. The volunteers used in the simulation tests were the authors of this paper, and the gender of the volunteers had no impact on the research results. The aforementioned tests did not require approval from an ethics committee.

## Declaration of competing interest

We declare that we have no financial and personal relationships with other people or organizations that can inappropriately influence our work, there is no professional or other personal interest of any nature or kind in any product, service and/or company that could be construed as influencing the position presented in, or the review of, the manuscript entitled “Multifunctional hydrogel sensors with dynamic covalent networks for machine learning-assisted Parkinson's disease diagnosis and encrypted human-computer interaction”.

## Data Availability

Data will be made available on request.
