# Supplementary material for: Multifunctional hydrogel sensors with dynamic covalent networks for machine learning-assisted Parkinson's disease diagnosis and encrypted human-computer interaction
Source: Mater Today Bio. 2025 Nov 4;35:102524. doi: 10.1016/j.mtbio.2025.102524 (PMC12639595; doi:10.1016/j.mtbio.2025.102524)
Supplement: Multimedia component 1 [file mmc1.docx]

Supporting Information

Multifunctional hydrogel sensors with dynamic covalent networks for machine learning-assisted Parkinson's disease diagnosis and encrypted human-computer interaction

Siqi Ding*^a1^,* Xiao Yu*^b1^,* Qi Wang*^c1^,* Peng Luo*^d1^,* Hua Li*^e^,* Zhengrui Li*^c^,* Ruhan Wang*^f^,* Hengrui Liu*^g^*,* Yucang He*^h^*,* Jinyao Nong*^e,i^*, and* Chao Zhang*^f^**

^a^ Department of Neurology, The Affiliated Yiwu Hospital of Wenzhou Medical University, 699 Jiangdong Road, Yiwu City, Zhejiang Province, 322000, People's Republic of China.

^b^ Department of Otolaryngology-Head and Neck Surgery, Shanghai Sixth People's Hospital Affiliated to Shanghai Jiao Tong University School of Medicine, Shanghai 200030, China.

^c^ Department of Oncology, Ruijin Hospital, Shanghai Jiao Tong University School of Medicine, Shanghai, 200025, China.

^d^ Southern Medical University, Guangzhou, Guangdong, China.

^e^ Life Science and Clinical Medicine Research Center, Affiliated Hospital of Youjiang Medical University for Nationalities, Baise, 533000, Guangxi, China.

^f^ Department of Neurosurgery, Qilu hospital of shandong university.

^g^ Department of Biochemistry, University Of Cambridge, Cambridge, UK.

^h^ Department of Plastic Surgery, First Affiliated Hospital of Wenzhou Medical University, Nanbaixiang, Ouhai Direct, Wenzhou City, Zhejiang Province, 325000, China.

^i^ Department of Rehabilitation Medicine, Affiliated Hospital of Youjiang Medical University for Nationalities, Baise, 533000, Guangxi, China.

^1^These authors contributed equally to this work.

Corresponding authors: Hengrui Liu, Yucang He, Jinyao Nong, Chao Zhang

E-mails: hl546@cam.ac.uk(H.R. Liu), [heyucang0@163.com](mailto:heyucang0@163.com) (Y. C. He), [342023009@qq.com](mailto:342023009@qq.com) (J.Y. Nong), qlyy_zc@163.com (C. Zhang)

**Materials**

Methacrylic anhydride (MA), sodium lignosulfonate (LS), and methacrylated gelatin (GelMA) were purchased from Aladdin. Ammonium persulfate (APS), N,N'-Methylenebisacrylamide (MBA), Nylon6, and formic acid were sourced from Macklin. Carboxymethylcellulose (CMC), acrylamide (AM), 4-Dimethylaminopyridine (4-DMAP) and sodium periodate (NaIO_4_) were obtained from Bide Pharmatech. Deionized water was used throughout experiments.(Table S1)

**Preparation of MLS, OCMC**

MLS: 5 g of LS was dissolved in 50 g of EG and the mixture was sonicated for 30 minutes to form a completely homogeneous solution. 220 ml of methacrylic anhydride and 2.2 g of 4-DMAP were added to the solution and the reaction mixture was stirred at 60 °C for 24 h. After cooling to room temperature, the reaction mixture was poured into 1 L of isopropanol to precipitate lignosulfonate methacrylate (MLS). The precipitated product was centrifuged at 5000 rpm for 10 min to allow the precipitated product to settle. The precipitated MLS was transferred to a room temperature vacuum oven for 3 days to remove the remaining solvent. OCMC: 7 g of CMC is dissolved in 60 ml of deionized water, sodium periodate (7 g) is added to the solution and stirred at 35 °C for 5 h with a magnetic stirrer. The pH is adjusted to 2 using 1 M sulfuric acid and the resulting solution is magnetically stirred for an additional 24 h at 35 °C in the dark. OCMC was precipitated 96% by the addition of a large amount of ethanol, then recovered and washed thoroughly with a distilled water-ethanol solution.

**Preparation AGOM Hydrogel**

First, 4 g and 0.2 g of OCMC were added to 10 g of deionized water and stirred homogeneously for 30 min. Then 0.4 g of GelMA was added and continued stirring until dissolved, followed by different amounts of MLS (1 wt%, 2 wt%, 3 wt%, 4 wt%, and 5 wt%, Mass ratio of MLS to H_2_O). The mixture was stirred homogeneously for 2 hours. Afterwards, the solution was evacuated for 20 min to remove oxygen. Appropriate amounts of MBA and APS were added and the mixture was stirred in an ice bath with rapid stirring for 10 min. A hydrogel was formed after polymerization at 50 °C for 4 h，AA/OCMC/GelMA/MLSx, x is the mass ratio of MLS to H_2_O,We named it AGOMX hydrogel and similarly, PAA hydrogel, PAA/OCMC hydrogel and PAA/OCMC/GelMA hydrogel were prepared by the same process.

**Mechanical properties**

The tensile tests were performed using cylindrical samples (length 50mm, diameter 2mm) at a constant strain rate of 100 mm/min. For compression tests, cylindrical samples (height 40mm, diameter 15mm) were used with a loading rate of 5 mm/min. All tests were conducted five times, and the average values were calculated and reported as the final results.

**Characterization**

FT-IR spectra of dried adhesive samples were analyzed using a Fourier Transform Infrared (FTIR) spectrometer (Nicolet 560, USA). The spectral range of the measured wavenumbers spanned from 4000 to 400 cm^-^¹, with five measurements conducted for each sample. The sample's morphology was examined using a scanning electron microscope (SEM, Hitachi S-4800, Japan) operated at an accelerating voltage of 5 kV. Additionally, the crystal structure was characterized through powder X-ray diffraction (XRD) analysis.X-ray photoelectron spectroscopy (XPS) (ESCALAB 250Xi, USA) was employed to obtain the XPS spectra of the samples.

**Adhesion performance experiments**

The adhesion properties of hydrogels were evaluated on various substrates such as rubber, glass, metal, paper, wood and plastic. For testing, a 20 mm × 10 mm sample of hydrogel was placed between two substrate surfaces. Shear tensile tests were performed using a universal testing machine at a constant speed of 20 mm/min. Each experiment was repeated three times to ensure the reliability of the data.

The lap shear strength (τ) was calculated according to the following equation:

where *F_max_* represents the maximum applied load (N), and *S* is the contact area (m^2^) between the hydrogel and the substrates.


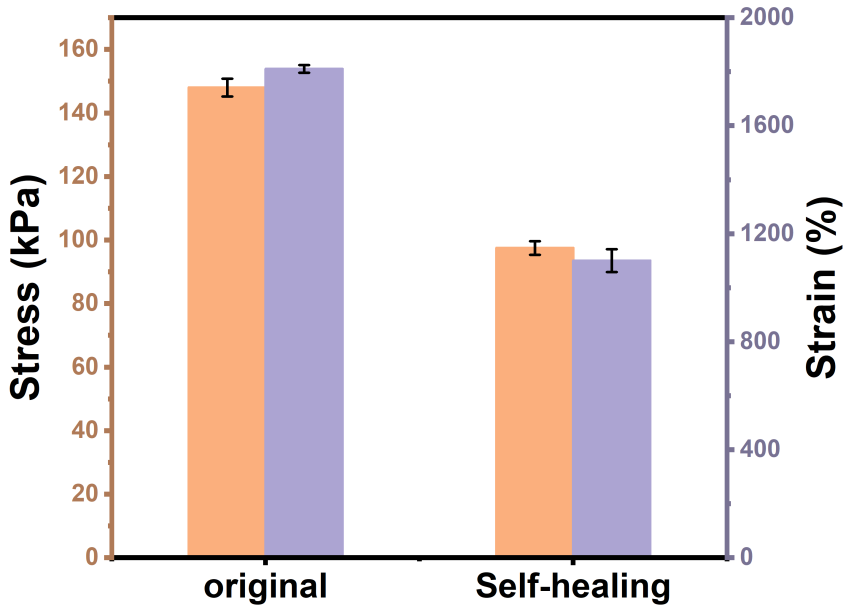


**Figure S1.** Mechanical properties and elongation of initial hydrogels and self-healing hydrogels.

**
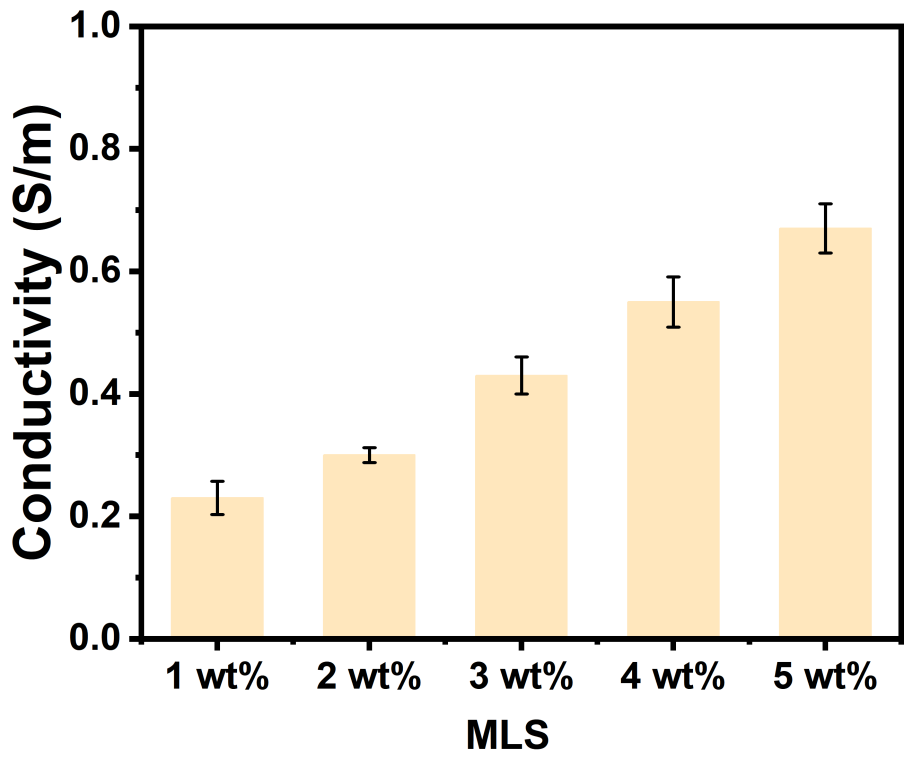
**

**Figure S2.** Conductivity of hydrogels prepared with different contents of MLS.

.

**Table S1.** The compositions of hydrogels.

| Material  Name | AA  (g) | OCMC  (g) | GelMA  (g) | MLS  (g) | MBA  (g) | APS  (g) | H_2_O  (g) |
| --- | --- | --- | --- | --- | --- | --- | --- |
| PAA  PAA/OCMC  PAA/OCMC/GelMA  PAA/OCMC/GelMA/MLS_1_  PAA/OCMC/GelMA/MLS_2_ | 4  4  4  4  4 | 0  0.2  0.2  0.2  0.2 | 0  0  0.2  0.2  0.2 | 0  0  0  0.01  0.02 | 0.004  0.004  0.004  0.004  0.004 | 0.04  0.04  0.04  0.04  0.04 | 10  10  10  10  10 |
| PAA/OCMC/GelMA/MLS_3_ | 4 | 0.2 | 0.2 | 0.03 | 0.004 | 0.04 | 10 |
| PAA/OCMC/GelMA/MLS_4_ | 4 | 0.2 | 0.2 | 0.04 | 0.004 | 0.04 | 10 |
| PAA/OCMC/GelMA/MLS_5_ | 4 | 0.2 | 0.2 | 0.05 | 0.004 | 0.04 | 10 |

**Table S2.** Performance comparison of the hydrogels with other previous reports.

| Materials | Tensile stress (MPa) | Tensile strain (%) | σ (S/m) | GF | Ref. |
| --- | --- | --- | --- | --- | --- |
| PA-PAAM-CS | 0.15 | 760 | 1.19 | 2.12 | [1] |
| PAM-OSA | 0.015 | 770 | 3.8 | 3.40 | [2] |
| P(AA-HEMA)-QCS-TA | 0.096 | 990 | / | 1.13 | [3] |
| P(SBMA-AAc-TA-PEI | 0.31 | 600 | 0.45 | 1.27 | [4] |
| MCC-PAA-AgNWs-CNTs | 0.45 | 558 | 0.045 | 4.73 | [5] |
| SSt-PAA-G | 0.09 | 719 | / | 4.2 | [6] |
| PAA-OCMC-GelMA-MLS | 0.146 | 1820 | 0.55 | 5.31 | This work |

**Supporting References**

[1] H. Zeng, H. Ma, L. Xu, J. Gao, M. Yan, Q. Wang, Tough, highly conductive and frost-resistant chitosan based hydrogel for flexible sensor, International Journal of Biological Macromolecules 297 (2025) 139847. https://doi.org/10.1016/j.ijbiomac.2025.139847.

[2] Y. Li, J. Zhu, L. Chen, N. Chen, X. Chen, J. Lv, Polysaccharide-driven self-healing dual-network hydrogel via Schiff base for high-performance flexible sensing, Carbohydrate Polymers 370 (2025) 124404. https://doi.org/10.1016/j.carbpol.2025.124404.

[3] R. Wu, T. Zhu, Y. Ni, C. Wu, W. Wang, K. Zhao, J. Huang, Y. Lai, UV‐Cured Dense Double Network Hydrogel via Multiple Dynamic Crosslinking for Stable Amphibious Motion Sensing, (n.d.). https://doi.org/10.1002/adfm.202515120.

[4] Y. Li, H. Liu, R. Ma, Q. Liu, S. Ma, N. Han, L.-B. Xing, Instant self-healing adhesive hydrogel sensors: dual-network design for real-time human motion tracking, J. Mater. Chem. A 13 (2025) 28152–28159. https://doi.org/10.1039/D5TA04564A.

[5] D. Wei, Y. Chen, S. Lv, J. Zuo, L. Liu, Y. Mu, J. Liu, J. Wang, One-step fabrication of dual-network cellulose-based hydrogel sensors with high flexibility and conductivity under ZnCl2 solvent method for flexible sensing properties, International Journal of Biological Macromolecules 295 (2025) 139440. https://doi.org/10.1016/j.ijbiomac.2024.139440.

[6] J. Liu, S. Lv, Y. Mu, D. Wei, Y. Chen, T. He, J. She, L. Liu, Flexible and wearable strain sensor based on SSt/PAA/G composite hydrogel for human–machine interaction applications, Chemical Engineering Journal 522 (2025) 167678. https://doi.org/10.1016/j.cej.2025.167678.
